# Changes in Academic Standardized Testing After Pediatric Intensive Care

**DOI:** 10.1001/jamanetworkopen.2026.9948

**Published:** 2026-04-30

**Authors:** Claire C. Foster, Melanie Boyd, Erin F. Carlton, Jill Fussell, Chayla R. Slaton, Sarah McKenzie, Katherine Irby, Peter M. Mourani, Clare C. Brown, Aline B. Maddux

**Affiliations:** 1 Department of Pediatrics, Division of Critical Care Medicine, University of Arkansas for Medical Sciences and Arkansas Children’s Hospital, Little Rock; 2 Department of Pediatrics, Division of Developmental and Behavioral Pediatrics, University of Arkansas for Medical Sciences and Arkansas Children’s Hospital, Little Rock; 3 Department of Health Policy and Management, University of Arkansas for Medical Sciences, Little Rock; 4 Department of Pediatrics, Division of Critical Care Medicine, University of Michigan, Ann Arbor; 5 Susan B. Meister Child Health Evaluation and Research Center, Department of Pediatrics, University of Michigan, Ann Arbor; 6 Department of Pediatrics, Division of Psychology, University of Arkansas for Medical Sciences, Little Rock; 7 Department of Education Reform, College of Education and Health Professions, University of Arkansas, Fayetteville; 8 Department of Pediatrics, Section of Critical Care Medicine, University of Colorado School of Medicine and Children’s Hospital Colorado, Aurora

## Abstract

**Importance:**

Children who survive critical illness commonly experience long-term morbidities. Little is known about the association of critical illness with cognitive health due to lack of preillness and postillness assessments and an adequate comparison population.

**Objective:**

To use school-based testing to evaluate cognitive health outcomes among children treated in the pediatric intensive care unit (PICU) compared with non–PICU-exposed control students.

**Design, Setting, and Participants:**

This retrospective case-control study used statewide academic data and propensity score matching of 1088 patients admitted to the only PICU in Arkansas from January 1, 2008, to December 31, 2018, as well as controls matched on sociodemographic and pre–PICU admission academic factors. Statistical analysis was performed from March 2024 to September 2025.

**Exposure:**

PICU admission.

**Main Outcomes and Measures:**

Primary outcomes were (1) return to standardized testing, assessed using multivariable logistic regression to assess the odds of having a standardized test within 2 years after PICU admission compared with controls, and (2) change in pre-PICU to post-PICU test scores for PICU patients compared with propensity-matched control students, assessed using multivariable linear regression.

**Results:**

In this case-control study of 1088 school-aged patients (mean [SD] age, 12.1 [1.6] years; 566 girls [52.0%]), fewer PICU patients than controls had test scores after admission for math (80.6% [874 of 1085] vs 86.5% [938 of 1085]; adjusted odds ratio [AOR], 0.64 [95% CI, 0.51-0.81]) and reading (81.1% [877 of 1081] vs 87.1% [941 of 1081]; AOR, 0.64 [95% CI, 0.51-0.82]). PICU patients’ preadmission *z* scores were below average in math (*z* = −0.23 [95% CI, −0.29 to −0.16]) and reading (*z* = −0.22 [95% CI, −0.29 to −0.15]) compared with Arkansas students in the same grade and year. In adjusted pre-post analyses, PICU patients had a small but significant decrease in reading relative to controls (−0.07 [95% CI, −0.14 to −0.01]). The change in math score was not statistically significant (−0.06 [95% CI, −0.13 to 0.003]).

**Conclusions and Relevance:**

This study suggests that PICU patients were less likely to take standardized tests after discharge and that those who did had greater decreases in reading scores relative to matched controls. Future studies should identify risk factors for nonreturn to testing and score decrease.

## Key Points

**Question:**

Is pediatric intensive care unit (PICU) admission associated with a decrease in academic achievement assessment scores?

**Findings:**

In this case-control study of 1088 PICU patients with assessment scores available, compared with controls, preadmission scores were lower than the state mean among students in the PICU, and their scores decreased more after PICU admission.

**Meaning:**

This study suggests that children treated in the PICU may benefit from comprehensive academic assessments and additional support on return to the classroom.

## Teaser

This case-control study uses hospital and statewide department of education data to evaluate changes in students’ performance on academic standardized tests after admission to the pediatric intensive care unit.

## Introduction

Mortality rates of critically ill children are below 2%, but children experiencing pediatric critical illness and injury often experience significant morbidities across physical, cognitive, emotional, and social health domains.^1,2,3,4^ The intersection of impairments across these 4 domains may be associated with developmental outcomes, with varying recovery trajectories. Within the domain of cognitive health, pediatric intensive care unit (PICU) admission is associated with a decrease in cognitive functioning.^5^ However, our understanding of the association of PICU admission with cognitive health is limited by a lack of preillness cognitive baseline data and the challenge of accounting for expected developmental trajectory.^6^ Previous studies have relied on comparisons with population means or sibling-based controls.^7,8,9^ Academic performance is an important and understudied component of pediatric cognitive health and has been used to evaluate other critically ill pediatric populations including neonatal intensive care unit patients.^10,11,12^ These studies found that extremely premature infants, infants with neonatal hypoglycemia, and infants with complex anomalies had lower scores than matched controls on standardized school-based assessments.

This study expands previous research by using standardized testing data collected by the Arkansas Department of Education to assess cognitive outcomes after a PICU admission. This statewide educational database provides opportunities for both preillness and postillness academic assessments and a robust control cohort for comparing expected performance. The aim of this study was to quantify the association of a PICU admission with academic achievement test participation and performance in mathematics and reading or language arts (henceforth, *reading*). We hypothesized that PICU patients would be less likely to return to standard achievement testing and that those who did return would demonstrate a decrease in mathematics and reading scores relative to a matched population of non–PICU-exposed children.

## Methods

Data for this case-control study were collected retrospectively and granted a waiver of informed consent and Health Insurance Portability and Accountability Act authorization by the institutional review board at the University of Arkansas for Medical Sciences. Educational data release was consistent with Family Educational Rights and Privacy Act disclosure laws.^13^ We adhered to the Strengthening the Reporting of Observational Studies in Epidemiology (STROBE) reporting guideline.

This retrospective case-control study was conducted at a quaternary children’s hospital with 26 PICU beds that serves as the only PICU in Arkansas. We identified PICU patients using the Virtual Pediatric System (VPS, LLC) database.^14^ Eligible patients survived the hospitalization that included their PICU admission, were aged 7 to 17 years at admission, and were admitted between January 1, 2008, through December 31, 2018. These dates capture all patients maintained in the VPS registry at our institution but end prior to the disruption in education and educational testing due to the COVID-19 pandemic. Patients who experienced multiple PICU admissions during the study period were included for their first eligible admission. Using an exact match based on first and last name and social security number, PICU patients were matched with standardized test information from the Arkansas Department of Education. Patients were excluded if they were unable to be linked with the Arkansas Department of Education data prior to admission, did not have complete educational data, were not administered a standard academic test during the 2 years preceding PICU admission, or were not in grades 3 to 7 at the time of PICU admission. Although all school-aged patients were included, grade-based exclusion criteria were established to limit the study population to students with the potential for both pre-PICU and post-PICU standardized assessments, which were administered to Arkansas students in grades 3 through 8. Students with standardized test scores who were not identified as having a PICU admission were eligible to be controls.

Our primary study outcomes were (1) return to standardized testing, defined by presence of a post-PICU math or reading test score within 2 years of PICU admission, and (2) change in math and reading scores from pre-PICU to post-PICU admission. Multiple versions of standardized tests were administered during the study period. To facilitate comparison across test versions, grade levels, and study years, standardized test scores were converted to statewide *z* scores for each year, test subject, and grade level. April 1 of each year was chosen as a standardized test administration date.

In addition to standardized test scores, we also extracted school, gender, race and ethnicity, presence of an educational accommodation (ie, an Individualized Educational Program [IEP] or Section 504 plan), homelessness, English-learner status, and eligibility for free or reduced-price lunch through the National School Lunch Program from the Arkansas Department of Education data.^15^ A student, parent, or guardian reported gender (male, female) and race and ethnicity to the Arkansas Department of Education. The education data recorded racial and ethnic categories of Asian, Black or African American, Hispanic, Native American or Alaska Native, Native Hawaiian or Pacific Islander, White and 2 or more races. For analyses, “other” included students who identified as Asian, Native American or Alaska Native, Native Hawaiian or Pacific Islander, and 2 or more races. We included race and ethnicity as covariates to account for the unmeasured systemic and structural factors that may influence educational outcomes.

For the control population, students were included as a potential match if the student had a standardized test score in the respective preadmission baseline year. To identify matched controls, we used propensity score matching to conduct a 1:1 nearest neighbor match with replacement and a caliper of 0.2. We used exact matching on school, grade level, year, and baseline test score quintile, and we used gender, race and ethnicity, homelessness status, free or reduced-price lunch status, and English language learner status to calculate propensity scores. Four separate propensity score matches were conducted, 1 for each outcome and test subject. We examined characteristics of each match for covariate balance using standardized mean differences (≤0.1) to confirm adequate match.

### Statistical Analysis

Statistical analysis was performed from March 2024 to September 2025. To assess the proportion of children who remained alive and eligible for assessment (ie, within the Arkansas school system) during the post-PICU period, we assessed the frequency of identification of patients and controls in the educational database during the post-PICU period. We used multivariable logistic regression to assess the odds of having a post-PICU test in the 2 years after PICU admission, adjusted for gender, race and ethnicity, IEP or Section 504 plan, English language learner status, and school-collected surrogates of socioeconomic status including homelessness and free or reduced lunch status. Models were constructed separately for math and reading tests. The primary covariate of interest was PICU exposure.

For patients with pre-PICU and post-PICU test scores available, we used multivariable linear regression with a pre-post design with a matched control. The primary coefficient of interest was the interaction between the post variable and the PICU exposure variable, which characterizes the predifference to postdifference for a patient admitted to the PICU vs the patient’s matched control. This allows the change for non–PICU-admitted students to serve as the counterfactual, or what we would expect the change to be for a patient if that patient had not had a PICU admission.

We conducted an ad hoc analysis to test for an association between duration of PICU admission and both primary outcomes. Patients admitted for less than 1 day were compared with patients admitted for 1 to 6 days and for 7 or more days using an analysis of variance. We also evaluated time between PICU discharge and post-PICU test, categorized as less than 4 months (approximating discharge in the same semester as testing), 4 to 11 months (discharge in prior semester), and 12 to 24 months (discharge in the prior year) using an analysis of variance. We conducted a sensitivity analysis of patient-control dyads who were in the same schools after PICU discharge to remove potential variation due to educational environment. All *P* values were from 2-sided tests and results were deemed statistically significant at *P* < .05. No data were imputed and missing data were reported. Analyses were conducted using SAS software, version 9.4 (SAS Institute Inc).

## Results

We identified 3118 unique patients meeting inclusion criteria using the Virtual Pediatric System database, of whom 2755 (88.4%) were matched in the Arkansas Department of Education Database. Of these, 1667 patients were excluded, most often due to lack of preadmission test or age, resulting in 1088 study cohort patients (mean [SD] age, 12.1 [1.6] years; 566 girls [52.0%] and 522 boys [48.0%]; 279 Black or African American [25.6%], 83 Hispanic [7.6%], 678 White [62.3%], and 48 other race or ethnicity [4.4%]) (Figure; Table 1). Of these 1088 PICU patients, 1085 (99.7%) with preadmission math assessment scores and 1081 (99.4%) with preadmission reading assessment scores were matched with controls. More than 95% of the patients and controls were identified in the education database during the post-PICU period and the proportions of patients and control students missing from the post-PICU period data were low across all study groups: for math, 4.9% of patients admitted to the PICU (53 of 1085) and 3.1% of control students (34 of 1085) were not identified and, for reading, 5.0% of patients admitted to the PICU (54 of 1081) and 3.3% of controls (36 of 1081) were not identified. Characteristics of patients and controls were summarized for the binary return to testing analysis (eTable 1 in Supplement 1).

**Figure.**
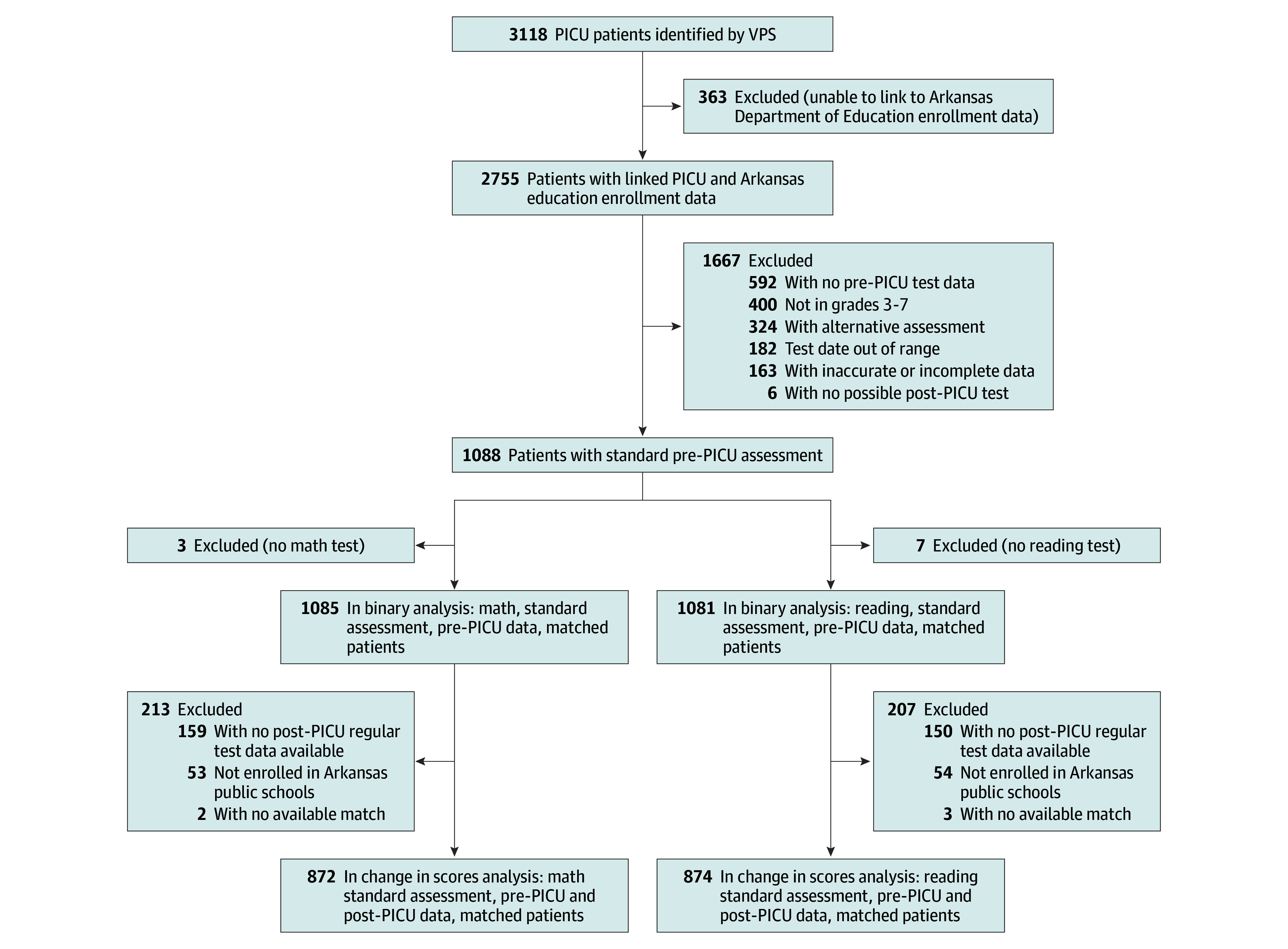
Enrollment Diagram Analyses conducted using data from the Arkansas Department of Education and Arkansas Children’s Hospital Virtual Pediatric Systems (VPS, LLC) database. PICU indicates pediatric intensive care unit.

**Table 1.** Demographic Characteristics of PICU Patients Eligible for Matching^a^

Characteristic	Patients, No. (%)(N = 1088)
Grade prior to PICU admission^b^	
3	178 (16.4)
4	198 (18.2)
5	202 (18.6)
6	228 (21.0)
7	282 (25.9)
Gender^c^	
Male	522 (48.0)
Female	566 (52.0)
Race and ethnicity^b^^,^^c^	
Black or African American	279 (25.6)
Hispanic	83 (7.6)
White	678 (62.3)
Other^d^	48 (4.4)
Social determinants of health^e^	
English language learner	51 (4.7)
Free and reduced-price lunch	719 (66.1)
Homelessness	31 (2.9)
Section 504 plan or IEP in place	305 (28.0)

Abbreviations: IEP, Individualized Education Program; PICU, pediatric intensive care unit.

^a^ Analyses were conducted using data from the Arkansas Department of Education and Arkansas Children’s Hospital Virtual Pediatric System database. Characteristics represent data from the year of test data associated with pre-PICU admission period.

^b^ Percentages not adding to 100 are due to rounding.

^c^ Categories were based on collected self-reported data from the Arkansas Department of Education.

^d^ Includes students identified as Asian, Native American or Alaska Native, Native Hawaiian or Pacific Islander, or 2 or more races.

^e^ Characteristics were collected from the Arkansas Department of Education database and used to represent aspects of social determinants of health.

Of the PICU patients with a preadmission math assessment score, 80.6% (874 of 1085) had a math score during the 2 years after PICU admission compared with 86.5% of controls (938 of 1085) (adjusted odds ratio [AOR], 0.64 [95% CI, 0.51-0.81]; *P* < .001) (Table 2). Of the PICU patients with preadmission reading test scores, 81.1% (877 of 1081) had a score available in the 2 years after PICU admission, compared with 87.1% of matched controls (941 of 1081) (AOR, 0.64 [95% CI, 0.51-0.82]; *P* < .001).

**Table 2.** Association Between PICU Admission and Return to Standard Assessment^a^

Domain	Students with standard post-PICU assessment, No./total No. (%)		OR (95% CI)	
	Controls	PICU patients	Unadjusted)^b^	Adjusted^c^
Math	938/1085 (86.5)	874/1085 (80.6)	0.65 (0.52-0.82)	0.64 (0.51-0.81)
Reading	941/1081 (87.1)	877/1081 (81.1)	0.64 (0.51-0.81)	0.64 (0.51-0.82)

Abbreviations: OR, odds ratio; PICU, pediatric intensive care unit.

^a^ Analyses conducted using data from the Arkansas Department of Education and Arkansas Children’s Hospital Virtual Pediatric System database.

^b^ Odds of having a postdischarge score relative to matched control.

^c^ Odds of having a postdischarge score relative to matched control, adjusted for gender, race and ethnicity, homelessness status, free or reduced-price lunch status, Individualized Education Program or Section 504 plan, and English language learner status.

Next, we evaluated PICU patients and matched controls who had both preadmission and postadmission scores available in math (n = 872) and reading (n = 874). Characteristics of these patients and controls are summarized in eTable 2 in Supplement 1. Preadmission *z* scores were lower than the mean for Arkansas students in the same grade and year for math (−0.23 [95% CI, −0.29 to −0.16]) and reading (−0.22 [95% CI, −0.29 to −0.15]) (Table 3).

**Table 3.** Association Between PICU Admission and Standardized Assessment Scores for Math and Reading^a^

Test domain	*z *Score (95% CI)				Change in PICU patients’ scores (95% CI)	
	PICU patients		Controls			
	Preadmission^b^	Postadmission^c^	Preadmission^b^	Postadmission^c^	Unadjusted coefficient^d^	Adjusted coefficient^e^
Math	−0.23 (−0.29 to −0.16)	−0.26 (−0.33 to −0.20)	−0.19 (−0.25 to −0.12)	−0.16 (−0.23 to −0.10)	−0.06 (−0.13 to −0.001)	−0.06 (−0.13 to 0.003)
Reading	−0.22 (−0.29 to −0.15)	−0.26 (−0.32 to −0.19)	−0.16 (−0.23 to −0.10)	−0.12 (−0.19 to −0.05)	−0.08 (−0.14 to −0.02)	−0.07 (−0.14 to −0.01)

Abbreviation: PICU, pediatric intensive care unit.

^a^ Analyses conducted using data from the Arkansas Department of Education and Arkansas Children’s Hospital Virtual Pediatric System database.

^b^ Most proximal test score within 2 years prior to PICU admission.

^c^ Most proximal test score within 2 years after admission.

^d^ Change in score of PICU patients before discharge to after discharge compared with change in scores of matched controls.

^e^ Change in score of PICU patients before discharge to after discharge compared with change in scores of matched controls adjusted for gender, race and ethnicity, homelessness status, free or reduced-price lunch status, Individualized Education Program or Section 504 plan, and English language learner status.

In unadjusted analyses, math scores of PICU patients decreased from −0.23 (95% CI, −0.29 to −0.16) to −0.26 (95% CI, −0.33 to −0.20), compared with an increase in scores for matched controls, from −0.19 (95% CI, −0.25 to −0.12) to −0.16 (95% CI, −0.23 to −0.10) (Table 3). Similarly, reading scores of PICU patients decreased from −0.22 (95% CI, −0.29 to −0.15) to −0.26 (95% CI, −0.32 to −0.19), compared with an increase in scores for matched controls, from −0.16 (95% CI, −0.23 to −0.10) to −0.12 (95% CI, −0.19 to −0.05). In unadjusted analyses to assess the change in pre-PICU to post-PICU admission scores for PICU patients vs controls, scores decreased for math and reading among PICU patients compared with controls (math, −0.06 [95% CI −0.13 to −0.001]; *P* = .047; and reading, −0.08 [95% CI, −0.14 to −0.02]; *P* = .01). In adjusted pre-post analyses, there was a decrease in reading scores (−0.07 [95% CI, −0.14 to −0.01]; *P* = .02). The change in math score was not statistically significant (−0.06 [95% CI, −0.13 to 0.003]; *P* = .06).

In ad hoc analyses, we evaluated the association between PICU length of stay and likelihood of returning to testing. Patients with stays of at least 7 days compared with 1 to 6 days and less than 1 day were less likely to return to testing for math (≥7 days, 71.9% [64 of 89]; 1-6 days, 78.2% [358 of 458]; <1 day, 84.0% [450 of 536]; *P* = .007) and reading (≥7 days, 71.9% [64 of 89]; 1-6 days, 79.1% [359 of 454]; <1 day, 84.3% [452 of 536]; *P* = .008) (Table 4). For patients who returned to testing after PICU admission, the decrease in *z* scores from the pre-PICU period to post-PICU period for math was greater among patients with longer stays: −0.39 (0.88) SDs for patients admitted for 7 days or more compared with −0.01 (0.75) SDs for patients admitted 1 to 6 days and −0.01 (0.66) SDs for patients admitted less than 1 day (*P* < .001) (Table 4). There was not a significant difference in reading scores across patients with differing lengths of stay (≥7 days, –0.20 [0.89]; 1-6 days, –0.04 [0.70]; <1 day, –0.02 [0.64]).

**Table 4.** Association Between Educational Outcomes and Length of Stay for PICU Patients^a^

Test domain	PICU length of stay			*P* value^b^
	<1 d	1-6 d	≥7 d	
**Postdischarge standardized assessment, No./total No. (%)**				
Math	450/536 (84.0)	358/458 (78.2)	64/89 (71.9)	.007
Reading	452/536 (84.3)	359/454 (79.1)	64/89 (71.9)	.008
**Change in *z* score (SD)**				
Math	−0.01 (0.66)	−0.01 (0.75)	−0.39 (0.88)	<.001
Reading	−0.02 (0.64)	−0.04 (0.70)	−0.20 (0.89)	.14

Abbreviation: PICU, pediatric intensive care unit.

^a^ Analyses conducted using data from the Arkansas Department of Education and Arkansas Children’s Hospital Virtual Pediatric System database.

^b^ Analyzed using an analysis of variance test.

In addition, we evaluated the time between PICU discharge and testing. There was no difference in the mean decrease in math or reading scores based on the time between PICU discharge and testing (eTable 3 in Supplement 1).

In the sensitivity analysis of patient-control dyads who remained in the same school during the post-PICU period, we analyzed 743 patient-control dyads with math assessments. For these 743 pairs, PICU patients experienced a nonsignificant decrease in scores compared with their matched control (−0.05 [95% CI, −0.12 to 0.03]; *P* = .21). Similarly, for 751 patient-control dyads with reading assessments, PICU patients demonstrated a nonsignificant decrease in scores relative to matched controls (−0.05 [95% CI, −0.12 to 0.01]; *P* = .12).

## Discussion

This study evaluated scores on standardized end-of-year school assessments to test for an association between PICU admission and academic performance scores among Arkansas children enrolled in grades 3 through 7 at the time of PICU admission. We found that patients had lower than mean scores prior to PICU admission, were less likely to return to standardized testing, and experienced decreases in their reading scores compared with their preadmission baseline, after adjusting for socioeconomic factors and preadmission need for educational support. Furthermore, these decreases were larger among patients with longer PICU stays. These changes emphasize the importance of understanding the association of PICU admission with cognitive functioning and school performance. In addition, identifying PICU patients at highest risk of decreases in performance will allow targeted anticipatory guidance to families and educators and development of programs aimed at improving the transition back to school to mitigate the risk of educational declines.

We identified that PICU patients’ scores were lower than the state mean in math and reading even prior to their PICU admission. Social disadvantage and chronic health conditions are associated with poorer school performance.^16,17^ Studies have demonstrated that children from disadvantaged backgrounds are overrepresented in PICU populations.^18,19,20,21^ In addition, children with medical complexity or chronic conditions account for most PICU admissions.^22,23^ We addressed these potential risk factors and the lower than mean pre-PICU scores using a robust matching process. By identifying a control student in the same educational setting, with similar demographic characteristics and test performance, immediately prior to a patient’s PICU admission, we were able to compare changes in return to testing and testing performance in a more meaningful way than comparing with statewide norms.

We found that 1 in 5 PICU patients did not return to standardized testing during the 2 years after their PICU admission. Federal education law, starting with the Elementary and Secondary Education Act in 1965, No Child Left Behind in 2002, and Every Student Succeeds Act of 2015, requires annual assessments in math and reading in grades 3 to 8 to improve instruction and monitor a child’s progress.^24,25^ Absence of results on standardized assessments after PICU admission may reflect either a decrease in cognitive functioning that required a change from standard assessments to alternative assessments or absence from school, preventing testing. There are many causes of school absence, and a recent American Academy of Pediatrics policy statement on school attendance details several causes of chronic absenteeism, defined as missing 15 days in a school year.^26^ Of PICU patients with acute respiratory failure, more than two-thirds of patients had subsequent absences from school and parents of these children reported their child missed a median of 16 days during the 6-month period after discharge.^27^ Other chronic conditions are also associated with increased absenteeism, including, but not limited to, diabetes, persistent fatigue, obesity, and asthma.^28,29,30,31^ Lack of participation in standard assessments leaves PICU patients at risk of underrepresentation when assessing instructional success and individual advancement.

Among patients who were able to participate in standardized testing after discharge, PICU patients with post-PICU test scores had lower scores relative to matched controls, although only decreases in reading remained significant in adjusted analyses. PICU patients with longer stays demonstrated greater decreases in post-PICU math scores, suggesting that a subset of patients may be at higher risk for poorer school performance. Having a decrease in one domain is concerning, as education research suggests there is likely codevelopment between math and reading skills.^32,33,34^ Future studies should evaluate for clinical factors associated with decreases in academic performance and score trajectories.

PICU patients had lower than state mean preadmission math and reading scores, making the findings of continued score decreases particularly concerning. Traditionally, an effect size, or *z* score change, of 0.2 is considered a large and meaningful educational change.^35^ Among PICU patients with a length of stay exceeding 7 days, we observed *z* score reductions of 0.39 in math and 0.20 in reading, both of which met the threshold for clinically meaningful decreases. Educational research has shown that elementary test scores correlate with outcome metrics such as earnings in adulthood, college attendance, home ownership, and retirement savings.^36^ In addition, math and reading ability at 7 years of age is associated with adult socioeconomic status even after controlling for childhood socioeconomic status.^37^ Our findings of reduced academic performance suggest that PICU patients may be at increased risk for persistent and lasting deficits not just in educational achievement but in future measures of success and financial stability.

### Limitations

This study had important limitations. Generalizability was limited by inclusion of only students in grades 3 through 7, those who took standardized assessments, and students enrolled in the Arkansas public school system. Restriction to those eligible for standardized testing excludes a large proportion of PICU patients who take alternative tests due to learning difficulties or cognitive deficits or those untested due to young age.^4^ Other methods are required to evaluate educational success among these patients. Because this study is limited to children in Arkansas Department of Education records, some patients or controls could have been missing due to relocation out of state or to private schools; however, our data demonstrated that this occurred in less than 5% of patients and controls. We were unable to characterize comorbidities as these data were not available for non–PICU-exposed control students. Demographic categories were limited to those maintained by the Department of Education. In addition, our databases lacked data delineating other PICS-p (post–intensive care syndrome in pediatrics) domains (eg, health-related quality of life). Our sample size was dictated by a single-center design and may not have been sufficient to detect differences. PICU patients and controls could have had a PICU stay prior to 2008 or at other PICUs; however, as the state’s only PICU, this was likely to be limited. Finally, cognitive health is complex and attainment of grade level skills, as measured by academic assessment, does not reflect the breadth of cognitive health that is critical for childhood development.

## Conclusions

In this case-control study, we found that children with a PICU admission were less likely to return to standardized testing than their peers and that patients who returned to testing experienced greater decreases in their scores, specifically in reading. Further research is necessary to identify PICU subgroups most at risk of a decrease in performance and the durability of the decreases.

## Notes

Supplement 1. **eTable 1.** Standardized Differences in Matched Cohorts, Return to Testing Analysis **eTable 2.** Standardized Differences in Matched Cohorts, Change in Pre- to Post-Score Analysis **eTable 3.** Change in Z-score by Test Domain and Months Between Admission and Test Administration

Supplement 2.
Data Sharing Statement
