# Supplementary material for: Changes in Academic Standardized Testing After Pediatric Intensive Care
Source: JAMA Netw Open. 2026 Apr 30;9(4):e269948. doi: 10.1001/jamanetworkopen.2026.9948 (PMC13133690; doi:10.1001/jamanetworkopen.2026.9948)
Supplement: Supplement 1. — eTable 1. Standardized Differences in Matched Cohorts, Return to Testing Analysis eTable 2. Standardized Differences in Matched Cohorts, Change in Pre- to Post-Score Analysis eTable 3. Change in Z-score by Test Domain and Months Between Admission and Test Administration [file jamanetwopen-e269948-s001.pdf]

Supplementary Online Content

Foster CC, Boyd M, Carlton EF, et al. Changes in academic standardized testing after pediatric intensive care. *JAMA Netw Open.* 2026;9(4):e269948. doi:10.1001/jamanetworkopen.2026.9948

eTable 1. Standardized Differences in Matched Cohorts, Return to Testing Analysis
eTable 2. Standardized Differences in Matched Cohorts, Change in Pre- to Post-Score Analysis
eTable 3. Change in Z-score by Test Domain and Months Between Admission and Test Administration

This supplementary material has been provided by the authors to give readers additional information about their work.

© 2026 Foster CC et al. *JAMA Network Open.*

eTable 1. Standardized Differences in Matched Cohorts, Return to Testing Analysis

	Math			Reading		
**Characteristic, n (%)**	**PICU patients n=1,085 (%)**	**Controls n=1,085 (%)**	***SD***	**PICU patients n=1,081 (%)**	**Controls n=1,081 (%)**	***SD***
**Gender, male**	520 (47.9)	519 (47.8)	0.002	521 (48.2)	516 (47.7)	0.009
**Race[a]**						
**Black/African American**	279 (25.7)	282 (26.0)	0.01	278 (25.7)	279 (25.8)	0.002
**Hispanic**	83 (7.7)	62 (5.7)	0.07	82 (7.6)	68 (6.3)	0.04
**White**	676 (62.3)	710 (65.4)	0.06	673 (62.3)	703 (65.0)	0.06
**Other [b]**	47 (4.3)	31 (2.9)	0.07	48 (4.4)	31 (2.9)	0.08
**Social Determinants of Health[c]**						
**English Language Learner**	51 (4.7)	46 (4.2)	0.02	51 (4.7)	49 (4.5)	0.02
**Free & Reduced Lunch**	718 (66.2)	730 (67.3)	0.02	716 (66.2)	725 (67.1)	0.01
**Homelessness**	31 (2.9)	18 (1.7)	0.08	31 (2.9)	21 (1.9)	0.07

Abbreviations: PICU, Pediatric Intensive Care Unit; SD, Standardized Difference
Note: Standardized differences provided for all variables in the match that were not an exact match.
[a]Categories were based on collected self-reported data from the Arkansas Department of Education . [b] "Other" includes students identified as Asian, Native American/Alaskan Native, Native Hawaiian/Pacific Islander, or Two or More Races [c]Characteristics were collected from the Arkansas Department of Education database and used to represent aspects of social determinants of health.
Analyses conducted using data from the Arkansas Department of Education and Arkansas Children's Hospital Virtual Pediatric System (VPS, LLC) database.

© 2026 Foster CC et al. *JAMA Network Open.*

eTable 2. Standardized Differences in Matched Cohorts, Change in Pre- to Post-Score Analysis

	**Math**			**Reading**		
**Characteristic, n (%)**	**PICU patients, n=872**	**Controls, n=872**	***SD***	**PICU patients[a], n=874**	**Controls, n=874**	***SD***
**Gender, male**	411 (47.1)	407 (46.7)	0.0009	415 (47.5)	417 (47.7)	0.005
**Race[b]**						
**Black/African American**	237 (27.2)	242 (27.8)	0.01	239 (27.4)	242 (27.7)	0.008
**Hispanic**	74 (8.5)	62 (7.1)	0.05	74 (8.5)	61 (7.0)	0.05
**White**	526 (60.3)	546 (62.6)	0.05	526 (60.2)	544 (62.2)	0.04
**Other[c]**	35 (4.0)	22 (2.5)	0.08	35 (4.0)	27 (3.1)	0.05
**Social Determinants of Health[d]**						
**Homelessness**	20 (2.3)	12 (1.4)	0.06	19 (2.2)	17 (2.0)	0.02
**Free & Reduced Lunch**	574 (65.8)	581 (66.6)	0.17	575 (65.8)	570 (65.2)	0.01
**English Language Learner**	44 (5.1)	38 (4.4)	0.03	44 (5.0)	38 (4.4)	0.03

Abbreviation: PICU, Pediatric Intensive Care Unit; SD, Standardized Difference
Note: Standardized differences provided for all variables in the match that were not an exact match.
[a]Percents not adding to 100 are due to rounding. [b]Categories were based on collected self-reported data from the Arkansas Department of Education . [c]"Other" includes students identified as Asian, Native American/Alaskan Native, Native Hawaiian/Pacific Islander, or Two or More Races [d]Characteristics were collected from the Arkansas Department of Education database and used to represent aspects of social determinants of health. Analyses conducted using data from the Arkansas Department of Education and Arkansas Children's Hospital Virtual Pediatric System (VPS, LLC) database.
Analyses conducted using data from the Arkansas Department of Education and Arkansas Children's Hospital Virtual Pediatric System (VPS, LLC) database.

© 2026 Foster CC et al. *JAMA Network Open.*

eTable 3. Change in Z-score by Test Domain and Months Between Admission and Test Administration

Test Domain	<4 Months Between Change in Z-score	4-11 Months Between Change in Z-score	12-24 Months Between Change in Z-score	p-value[a]
**Math**	-0.11	-0.009	-0.04	p=0.22
**Reading**	-0.1	-0.02	-0.02	p=0.33

Abbreviations: PICU: Pediatric Intensive Care Unit.
[a]Analyzed using an analysis of variance test.
Analyses conducted using data from the Arkansas Department of Education and Arkansas Children's Hospital Virtual Pediatric System (VPS, LLC) database.

© 2026 Foster CC et al. *JAMA Network Open.*
