# Supplementary material for: Changes in Academic Standardized Testing After Pediatric Intensive Care
Source: JAMA Netw Open. 2026 Apr 30;9(4):e269948. doi: 10.1001/jamanetworkopen.2026.9948 (PMC13133690; doi:10.1001/jamanetworkopen.2026.9948)
Supplement: Supplement 2. — Data Sharing Statement [file jamanetwopen-e269948-s002.pdf]

Data Sharing Statement

Foster. Changes in Academic Standardized Testing After Pediatric Intensive Care. *JAMA Netw Open*. Published April 30, 2026. doi:10.1001/jamanetworkopen.2026.9948

Data
Data available: No
